# Comparative ecological and behavioral study of *Macaca assamensis* and *M. mulatta* in Shivapuri Nagarjun National Park, Nepal

**DOI:** 10.1007/s10329-020-00810-9

**Published:** 2020-03-16

**Authors:** Sunil Khatiwada, Pavan Kumar Paudel, Mukesh K. Chalise, Hideshi Ogawa

**Affiliations:** 1 Department of Zoology, Tri-Chandra Multiple Campus, Tribhuvan University, Kirtipur, Nepal; 2 Central Department of Zoology, Institute of Science and Technology, Tribhuvan University, Kirtipur, Nepal; 3 Nepal Biodiversity Research Society, Lalitpur, Nepal; 4 Faculty of Liberal Arts and Sciences, Chukyo University, Nagoya, Japan; 5 Department of Biology, University of North Carolina at Chapel Hill, Chapel Hill, USA

**Keywords:** Assamese macaque, Rhesus macaque, Shivapuri Nagarjun National Park (SNNP), Socioecological behavior, Sympatric

## Abstract

Resource partitioning reduces the competition between different species within the same habitat, promoting their coexistence. To understand how such species co-adapt to reduce conflicts, we examined the behavior of two primates, Assamese macaque (*Macaca assamensis*) and rhesus macaque (*Macaca mulatta*), from April 2017 to March 2018 in Sivapuri Nagarjun National Park (SNNP), Kathmandu Valley, Nepal. We performed 1580 and 1261 scan sessions on wild multi-male/multi-female groups of Assamese and rhesus macaques, respectively, at 15-min sampling intervals. Assamese macaques consumed fewer plant species (38 species) than rhesus macaques (88 species). Overlapping food sources between the macaque species resulted in a Pianka index of 0.5. Assamese macaques consumed more items of tree, climber, and vine species, whereas rhesus macaques fed on more shrub, herb, and grass species. The proportions of plant parts consumed by the two species differed—more leaves, fruits and cones were used by Assamese macaques than rhesus macaques, whereas more flowers, seeds, and pods were consumed by rhesus macaques than Assamese macaques. Assamese macaques had a smaller home range (0.55 km^2^) than rhesus macaques (4.23 km^2^), and Assamese macaques had a shorter daily moving distance (1.6 km) than rhesus macaques (4.0 km). Although feeding time did not differ between the two macaque species, less time was devoted to social activities by Assamese macaques (16.0%) than by rhesus macaque**s** (33.7%). Assamese macaques were generally arboreal, with 94.0% of their activities in trees, whereas rhesus macaques were largely terrestrial, with 58.5% of their activities on the ground. These differences in food selection, home-range size, ranging and activity patterns, and habitat use suggest that Assamese and rhesus macaques reduce resource competition through resource partitioning to coexist in a landscape matrix.

## Introduction

Many studies of sympatric and non-sympatric primates have indicated that diet and space are factors affecting competition and conflict. Several studies on primate populations have shown that primates adopt various strategies for coexistence—for example, Snodderly et al. (2019) reported that four sympatric species, *Ateles belzebuth, Lagothrix lagotricha poeppigii, Plecturocebus discolor,* and *Pithecia aequatorialis*, adopted temporal niche partitioning by feeding at different diurnal periods. Competitive food exclusivity in Diana monkeys (*Cercopithecus diana*) maintained resource partitioning by allowing access to fruit-rich diets through competitive exclusion of sympatric congeners (Kane and McGraw 2017). Mountain gorillas (*Gorilla beringei beringei*) showed variation in foraging and avoided competition by shifting their daily movements to largely mutually exclusive core areas in wild non-territorial groups to voluntarily avoid neighbors (Seiler et al. 2017). Similarly, a sympatric community, consisting of *Macaca leonina, Macaca mulatta, Trachypithecus pileatus,* and *Hylobates hoolock,* avoided conflict via different resource partitioning mechanisms such as food preference and selective utilization of forest strata, ranging patterns, and activity budgets (Feeroz 2011). Selection of different strategy may be contextual, depending on various factors such as group size, presence of predators, and the area of potential habitat. Therefore, behavioral adaptation must play an important role in closely related sympatric species to encroach different niches.

Information on interspecific competition and niche separation among primate species is essential for understanding natural mechanisms of coexistence (Fleagle and Mittermeier 1980). Koenig and Borries (2001) and Vandercone et al. (2012) reported that fruit and seed consumption can cause competition between sympatric species. Sympatric strepsirrhine species in south-eastern Madagascar showed small dietary overlaps, in combination with other strategies such as social structure plasticity, habitat alteration, and diet switching, to cope with food shortages (Erhart et al. 2018). Thus, flexibility in diet, ranging, and activity patterns in sympatric primate species suggests a strategy to avoid conflict.

Several earlier studies of Assamese macaque (*Macaca assamensis*) and rhesus macaque (*Macaca mulatta*) in Nepal, separately confirmed conflicts between human and non-human primates (Chalise 2013; Chalise and Ghimire 1998; Chalise et al. 2013a, b; Jones-Engel et al. 2006; Paudel 2017). Assamese macaques are typical in the mid-hills of Nepal, whereas rhesus macaques habitat ranges from the Tarai plains to the mid-hills (Chalise 2013). These macaque species have similar body mass (Smith and Jungers 1997), and both are distributed in the same geographic habitats in Nepal and other parts of South and Southeast Asia (Zhang et al. 2002); however, there is considerable variation in their dietary structures (Chalise et al. 2013a, b; Koirala et al. 2017; Sengupta et al. 2014). Assamese macaques in Nepal differ morphologically (Molur et al. 2003) and genetically (Khanal et al. 2018) from other subspecies, i.e., eastern Assamese macaques (*M. assamensis assamensis*) and western Assamese macaques (*M. assamensis pelops*). For conservation and management, it is imperative to obtain detailed information (e.g., behavioral activity and coexistence with other primate species) on protected non-human primates (His Majesty’s Government of Nepal [HMGN] 1973). Therefore, one goal of the present study was to compare the feeding ecology, dietary overlap, behavior, and niche separation of these two non-human primates living within the same landscape.

As theorized by ecological character displacement (Stuart and Losos 2013), we anticipated that variation in feeding ecology has led the two macaques to occupy different strata of the forest habitat, thus reducing resource competition. Comparative ecological studies of these species are required to understand the dietary and behavioral flexibility among coexisting macaques that underpins adaptations and the mechanisms of coexistence. Therefore, we collected ecological data and compared (1) activity budgets, (2) diets, and (3) ranging patterns of these closely related primate species sharing the same landscape habitat with different physical, dietary, and behavioral traits, with the aims of (1) determining habitat use and resource exploitation, (2) examining the seasonality, diurnal variation, and age–sex difference in their activity budget and food dependence, and (3) understanding variation in ranging and feeding strategies between the two macaque species. The results of our investigation will be helpful in assessing how habitat complexity shapes primate behavior.

## Methods

### Study sites

This study was conducted from April 2017 to March 2018 at Shivapuri Nagarjun National Park (SNNP) (27.80° N, 85.39° E), which has an area of 159 km^2^, with the highest elevation at 2732 m. The area includes religious sites, army barracks (scattered within the area), and a convent. Human settlements are located around the boundary of the national park. The study area lies on the northern fringe of Kathmandu Valley. SNNP has two forest patches, Shivapuri and Nagarjun, which were a continuous landscape before anthropogenic activities led to fragmentation. Both forests share similar climatic and weather patterns with similar vegetation: *Schima wallichii* forest, mixed broadleaf forest, pine forest, and dry oak forest (Bhandari and Chalise 2014; Sigdel et al. 2005).

Two species of nonhuman primates, Assamese macaque and rhesus macaque, sympatrically inhabit the national park. Other fauna associated with the macaque habitat are orange-bellied Himalayan squirrel (*Dremomys lokriah*), Irrawaddy squirrel (*Callosciurus pygerythrus*), Chinese pangolin (*Manis pentadactyla*), Eurasian wild boar (*Sus scrofa*), common leopard (*Panthera pardus*), barking deer (*Muntiacus muntjak*), and sambar deer (*Cervus unicolor*) (Bhandari and Chalise 2014).

### Design and data collection

**Fig. 1:**
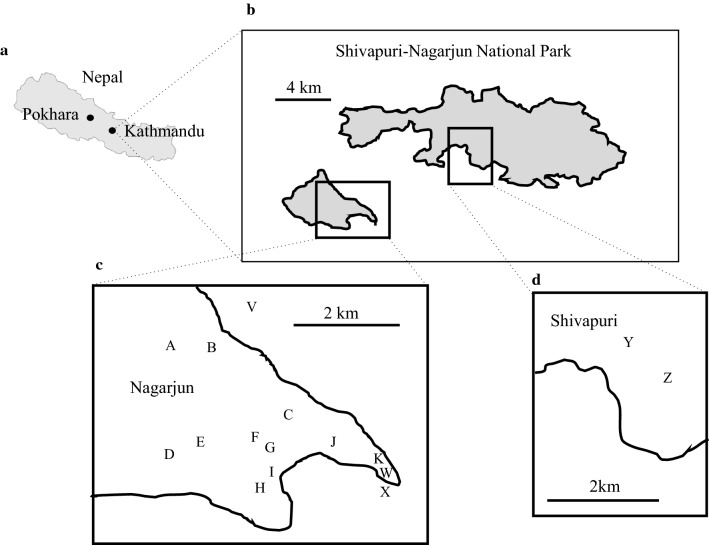
Distribution of social groups of rhesus and Assamese macaques in Shivapuri-Nagarjun National Park (SNNP). **a** Map of Nepal showing the location of the study site, SNNP, in the Kathmandu region. **b** Map of SNNP showing two forest patches, Shivapuri and Nagarjun. **c** The Nagarjun forest patch with the locations of social groups of Assamese macaques (A–K) and rhesus macaques (V–X). **d** The Shivapuri forest patch with the locations of social groups of rhesus macaques (Y and Z)

We selected one social group of rhesus macaque, the Rhesus Nagi (RN) group, in the Shivapuri forest and one social group of Assamese macaque, the Assamese Simpani (AS) group, in the Nagarjun forest (Fig. 1). Both groups inhabit the southern slope of the national park. The home ranges of these groups did not overlap. Although the home range of another group of rhesus macaques overlapped with that of Assamese macaques in the Nagarjun forest (Fig. 1), the group was too small for inclusion in this comparative study. Therefore, we selected the RN group in the Shivapuri forest.

The RN group was composed of 55 individuals, including 15 adult females, three adult males, three sub-adult males, nine juveniles, 15 infants, and ten newborns (newly born infants during the study period). A male group composed of 8–10 adult and sub-adult males sometimes followed the RN group; we did not collect data on this group because it did not intermix with the RN group. The AS group was composed of 30 individuals, including eight adult females, four adult males, one sub-adult male, six juveniles, three infants, six newborns (newly birthed infants during the study period), and two individuals of unknown age class. The study groups were accustomed to human presence. However, the early stages of our survey showed that the macaques spent most of their time in forests and did not gather in human territories. These macaques were exposed to human food only when they visited human settlements, such as the convent, army barracks, and houses bordering the national park, and during occasional encounters with visitors on hiking trails.

We searched and observed monkeys for approximately 90 h in each month from April 2017 to March 2018, and collected data for 395 and 315 h for the AS and RN groups, respectively. Before data collection, the macaques in the study groups were habituated to the presence of researchers. To bring uniformity to the observations, both observers (SK and PKP) observed the RN and AS groups together for 3 days in each site and confirmed behavior categories, tree heights, and age classes of the macaques. Subsequently, SK observed the RN group and PKP observed the AS group. The observers also met monthly in the study sites to confirm the accuracy of their data collection by observing the same subjects together. Our observations began early, at 07:00, and continued throughout the day, until 18:00, although the beginning and end times were not identical each day (mean ± SD observation time = 32.9 ± 7.5 h/month for the AS group; 26.0 ± 9.2 h/month for the RN group). We ended our observation when we could not follow the study group or lost the group and could not relocate it. We employed the same sampling method, scan sampling (Altmann 1974; Martin and Bateson 2007), for both study groups, with 5-min observations and 10-min intervals (inactivity period) in each (approx. mean ± SD observation time = 3.33 ± 1.3 h/day for the AS group; 2.8 ± 1.7 h/day for the RN group). Due to the complex landscape and thick vegetation, we could not observe all individuals during every session, hence we sampled as many individuals as possible during each session. We collected a total of 21,942 event data points from 1580 scan sessions for Assamese macaques (avg. ± SD = 11 ± 1.6 individuals per scan) and 23,059 event data points from 1261 scan sessions for rhesus macaques (avg. ± SD = 18 ± 6.7 individuals per scan) using binoculars.

Behavioral activities of the macaques were classified as feeding (eating food and foraging), resting (periods of inactivity, such as sleeping and chewing), moving, and social behavior (social grooming and other affiliative interactions, aggressive interactions, sexual interactions, and playing). When an individual showed two or more activities simultaneously, the most dominant activity was recorded, e.g., if an individual was engaged in playing and in chewing food, the activity was recorded as social behavior. The locations of the monkeys were recorded as ground or tree level, and approximate height (m) above the ground was recorded visually.

We used all-occurrence sampling to record new food items (Martin and Bateson 2007). We recorded the plant forms as tree, herb, shrub, grass, climber, vine, and orchid, and plant parts as fruit, cone, seed, pod, flower, leaf, stem, bark, nectar, sap, and whole plant. We referred to published studies (Sigdel et al. 2005; Koirala et al. 2017) for vegetation and plants in the study area to identify the types of fruiting and fodder plants. The plant leaves and fruits on which monkeys fed was photographed with a high-resolution camera and cross-referenced with herbarium samples. We finally identified most plants through collaboration with botanists from the Central Department of Botany, Tribhuvan University. Based on calorie and protein content, we divided plant parts into high-quality food (fruit, cone, seed, pod, flower, nectar, and sap) and low-quality food (leaf, stem, and bark). Insects were classified into high-quality food.

The Department of National Park and Wildlife Conservation, Nepal, provided permission to carry out fieldwork within the national park, and the study adhered to principles for ethical treatment of non-human primates.

### Data analysis

We made a list of food species identified throughout the year; plants consumed by the study groups were analyzed at monthly and seasonal scales to determine macaques’ dependence on different vegetation types throughout the year.

Niche overlap between the two species was calculated using the Pianka index (Pianka 1973):

$$O_{jk} = \frac{{\sum P_{ij} \cdot P_{ik} }}{{\sqrt {\left( {\sum P_{ij}^{2} \cdot \sum P_{ik}^{2} } \right)} }}$$

where *O*_*jk*_ is the overlap index between species *j* and *k*, and *P*_*ij*_ and *P*_*ik*_ are the percentages of utilization of resource category i (*i* = each feeding item) by species *j* and *k*, respectively. The percentage utilization of resources was determined by dividing the number of times a species used the plant by the total resource utilization throughout the year.

We summed the activity and locations of macaques from scan data and converted these into percentages. Activities and locations were categorized by season, time of day, and macaque age–sex classes. Scan data were analyzed using *χ*^2^ and Wilcoxon’s signed-rank tests.

Home-range sizes and moving distances were calculated for each month using tracks saved in GPS (Garmin Gpsmap 62). Tracks were extracted using the MapSource and imported into the QGIS ver. 3.2 to calculate home range. Tracks of > 6 h observations on the same day were used to calculate a daily moving distance. A heat-map was generated using QGIS based on a kernel density of 95% to determine the home range of each group for each season; other figures were prepared using Microsoft Excel ver. 2013. We used a *χ*^2^ test to compare dietary preferences, activity differences, and habitat utilization between macaque species. Wilcoxon’s signed-rank test was used to determine whether either of the studied species showed more wandering behavior and to examine feeding variation. The Mann–Whitney *U* test was used to examine differences in mobility between the two species.

## Results

### Activity

**Fig. 2:**
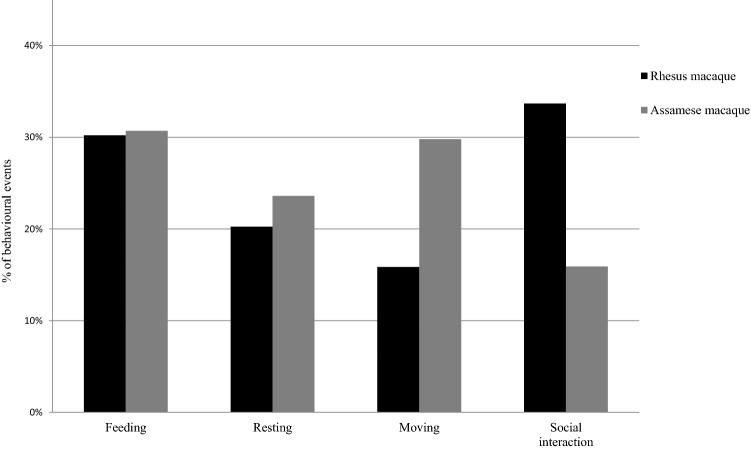
Percentages of activities in rhesus (left bars) and Assamese macaques (right bars)

The proportions of the four documented activities were different between the two species (*χ*^2^ test, *df* = 3, *χ*^2^ = 2441.1, *p* < 0.05). Assamese macaques spent most of the diurnal time in feeding (30.7%), whereas rhesus spent most time in social activities (33.7%). Less time was spent by Assamese macaques on social activities (16.0%) than by rhesus macaques (Fig. 2). The proportion of feeding was not significantly different between the two macaque species (*χ*^2^ test, *df* = 1, *χ*^2^ = 0.89, n.s. for feeding; *χ*^2^ = 57.8, *p* < 0.05 for resting; *χ*^2^ = 963.2, *p* < 0.05 for moving; *χ*^2^ = 1419.5, *p* < 0.05 for social interactions).

**Fig. 3:**
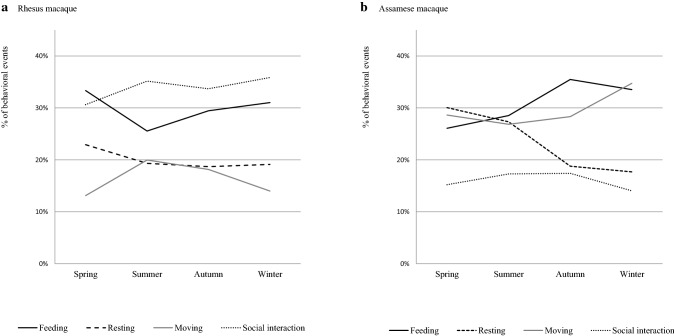
Seasonal changes in activities of rhesus and Assamese macaques. **a** Rhesus macaque. **b** Assamese macaque. Dark bold line represents feeding; bold broken line represents resting; light solid line represents moving; dotted line represents social interaction

Among four seasons, Assamese macaques spent the greatest proportion of their diurnal time feeding and engaging in social behavior in autumn (35.5 and 17.4%), resting in spring (30.1%), and moving in winter (34.7%), while rhesus macaques spent the greatest proportion of their diurnal time feeding and resting in spring (33.3 and 22.9%), moving in summer (20.0%), and engaging in social activities in winter (35.9%). Proportions of the four activities differed among seasons in both macaque species (Fig. 3; *χ*^2^ test, *df* = 9, *χ*^2^ = 246.2, 237, *p* < 0.05 in Assamese macaques; *χ*^2^ = 249.0, *p* < 0.05 in rhesus macaques). Among social activities, both macaque species performed mainly social grooming (77.4% in Assamese macaques and 50.5% in rhesus macaques), followed by aggressive and submissive behaviors (threat, fight, and passive submissive behavior; 14.6% in Assamese macaques and 26.6% in rhesus macaques).

**Fig. 4:**
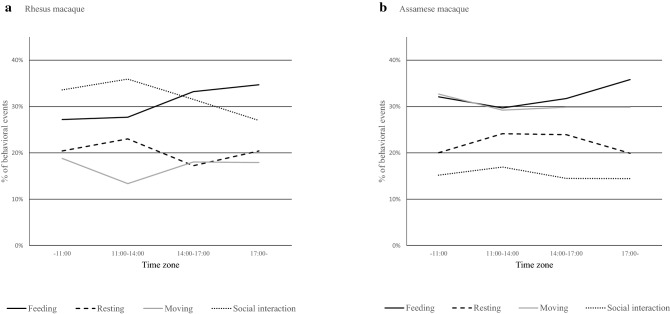
Diurnal variations in activities of rhesus and Assamese macaques. **a** Rhesus macaque. **b** Assamese macaque. Dark bold line represents feeding; bold broken line represents resting; light solid line represents moving; dotted line represents social interaction

There were significant associations between the time of day and activities in both macaque species (Fig. 4). Both macaques changed proportions of their activities from morning to evening (*χ*^2^ test, *df* = 9, *χ*^2^ = 47.04, *p* < 0.05 in Assamese macaques; *df* = 9, *χ*^2^ = 267.15, *p* < 0.05 in rhesus macaques).

**Fig. 5:**
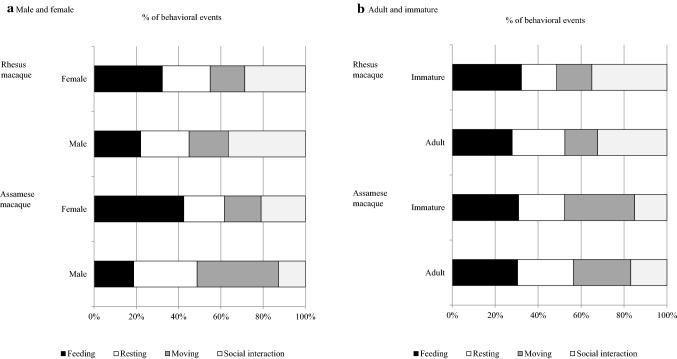
Comparison of percentages of activities between male and female macaques and between adult and immature macaques. **a** Male and female. **b** Adult and immature macaques. Dark shading represents feeding, white shading represents resting, dark-grey shading represents moving, and light-grey shading represents social interaction

Male Assamese macaques spent most of their time moving (38%), and females spent their time feeding (42.5%), whereas male rhesus macaques spent most of their time in social activities (36.5%), while females spent most of their time feeding (32.3%) (Fig. 5). Females of both macaque species spent more time feeding than males. Proportions of the four activities differed between males and females in both macaque species (*χ*^2^ test, *df* = 3, *χ*^2^ = 1743.3, *p* < 0.05 in Assamese macaques; *χ*^2^ = 183.5, *p* < 0.05 in rhesus macaques). In Assamese macaques, males spent more time moving than females (38.6 vs. 17.3%, respectively), less time feeding than females (18.7 vs. 42.5%), and less time in social interactions. In rhesus macaques, males spent more time moving than females (18.6 vs. 16.4%), more time in social interactions than females (36.5 vs. 28.7%), and less time feeding than females (22.1 vs. 32.3%). With regard to the age structure, the main activity of adult Assamese macaques was feeding (30.5%), and the least time was spent in social interaction (16.9%), whereas immature group members (infants, juveniles, and sub-adults) engaged predominantly in moving (32.6%) and spent less time in social activities (15.0%) (Fig. 5). In contrast, in rhesus macaques, both adults and immature members engaged mainly in social interactions (32.3 and 35.0%, respectively); adults engaged less in moving (15.3%), and the least frequent activities of immature members were moving and resting (16.4% each) (*χ*^2^ test, *df* = 3, *χ*^2^ = 240.27, *p* < 0.05) (Fig. 5).

### Feeding

Rhesus macaques utilized a total of 88 wild food species in 82 genera in the angiosperm and gymnosperm categories, whereas Assamese macaques consumed only 38 species in 29 genera (Tables 1 and 2). Among these foods, 17 plant genera were consumed by both macaque species. The Pianka index (niche overlap between two species for 1 year) was 0.5. Based on a feeding frequency > 10%, Assamese macaques consumed three plant species, whereas rhesus monkeys consumed one species (Table 1). There were significant differences in food selection and diet dependency between the two species. Rhesus macaques fed on more shrub, herb, and grass species, whereas Assamese macaques consumed more tree, climber, and vine species (*χ*^2^ test, *df* = 2, *χ*^2^ = 10.27, *p* < 0.05). Leaves, fruits and cones, and flowers accounted for 43.1, 34.5, and 12.1%, respectively, of the total number of food items consumed by Assamese macaques, and 35.0, 31.0, and 14.0% of those consumed by rhesus macaques (Table 3). Although both macaque species fed on various parts of plants in many forms, Assamese macaques showed less variety in their diet than did rhesus macaques (Table 3, Wilcoxon’s signed-rank test, *n* = number of plant forms = 7, *z* = − 2.20, *p* < 0.05; *n* = number of plant parts = 8, *z* = − 2.18, *p* < 0.05). Both macaque species fed on mosses (lichens), mushrooms, and leftover food at dump sites such as bread, biscuits, chocolate, soda, coriander, noodles, rice, chickpeas, canned food, flour, peas, peanuts, fruits (apple, mango, orange, banana, strawberry), and vegetables (green onion, garlic, radish, potato). Assamese macaques fed on spiders and on insects such as grasshoppers, caterpillars, and cocoons; these accounted for 29.8% of their diet. Both macaque species sometimes ate soil; rhesus macaques were seen licking cemented walls. Rhesus macaques consumed 31, 28, 42, and 44 plant species in summer, autumn, winter, and spring, whereas Assamese macaques consumed 24, 30, 16, and 29 plant species in these seasons, respectively (Table 1).

**Table 1:** Food species of rhesus and Assamese macaques in each month during scan sampling

No.		Species	Month												Number of month	Number of feeding records	% of feeding records	Life form	Parts eaten
			April	May	June	July	Aug.	Sept.	Oct.	Nov.	Dec.	Jan.	Feb.	March					
*Rhesus macaque*																			
1		*Aconogonum campanulatum*			+									+	2	3	0.1	Shrub	Stem
2		*Aconogonum mole*						+							1	1	0.0	Shrub	Fruit
3		*Albizia procera* (Roxb.) Benth.			+						+	+	+	+	5	105	3.5	Tree	Seed
4		*Alnus nepalensis*					+	+			+		+	+	5	16	0.5	Tree	Cone, leaf (petiole)
5		*Anisomeles indica* (L.) Kuntze							+					+	2	5	0.2	Herb	Flower
6		*Ardisia* sp.						+							1	2	0.1	Shrub	Leaf, fruit
7		*Bambuseae kunth*		+	+								+	+	4	43	1.4	Grass	Leaf
8		*Berberis aristate*	+									+		+	3	17	0.6	Shrub	Leaf, fruit
9		*Berberis* sp.	+	+											2	1	0.0	Shrub	Fruit
10		*Bidensa pilosa*			+		+	+	+		+	+	+	+	8	160	5.3	Herb	Flower, leaf
11		*Brassica juncea*	+	+							+	+	+	+	6	45	1.5	Herb	Leaf, stem
12		*Cannabis* sp.			+								+		2	6	0.2	Herb	Leaf
13		*Carex* sp.			+					+	+	+		+	5	15	0.5	Grass	Fruit
14		*Castanopsis indica*			+			+	+	+	+	+			6	132	4.3	Tree	Fruit
15		*Castanopsis tribuloides*	+		+					+	+			+	5	28	0.9	Tree	Fruit
16		*Celtis australis* L.	+		+			+							3	11	0.4	Tree	Leaf
17		*Chayote* sp.					+	+	+	+	+	+		+	7	175	5.8	Climber	Fruit, leaf, stem, tendril
18		*Choerospondias axillaris*			+			+			+	+	+	+	6	112	3.7	Tree	Fruit
19		*Cissampelos pareira*										+		+	2	1	0.0	Climber	Leaf
20		*Citrus limon*									+				1	1	0.0	Tree	Fruit
21		*Citrus maxima*											+		1	1	0.0	Tree	Fruit cover
22		*Codariocalyx motorius*										+			1	1	0.0	Herb	Flower
23		*Crotalatia cytisoides*					+								1	4	0.1	Shrub	Flower
24		*Cynodon dactylon*		+											1	3	0.1	Grass	Root
25		*Digitaria* sp.	+					+			+			+	4	38	1.2	Grass	Leaf, root
26		*Dioscorea* sp.						+							1	3	0.1	Climber	Fruit, leaf
27		*Equisetum arvense*									+				1	10	0.3	Herb	Plant
28		*Eriobotrya dubia* (Lindl.) Decne	+												1	7	0.2	Tree	Fruit
29		*Eurya acuminata* DC.	+	+			+							+	4	88	2.9	Tree	Flower, fruit, leaf
30		*Ficus glaberrina*	+	+	+							+	+	+	6	68	2.2	Tree	Fruit, leaf
31		*Gaultheria fragrantissima*		+			+								2	9	0.3	Shrub	Fruit
32		*Gladiolus* sp.					+								1	5	0.2	Shrub	Stem
33		*Globba clarkei* (Baker, Fl. Brit)						+							1	1	0.0	Herb	Flower
34		*Hedyotis* sp.	+	+			+		+	+	+	+	+	+	9	141	4.6	Herb	Leaf
35		*Indigofera exilis*										+			1	1	0.0	Shrub	Seed
36		*Lantana camara*					+					+	+		3	34	1.1	Shrub	Leaf, fruit
37		*Leucosceptrum canum*											+		1	9	0.3	Tree	Flower
38		*Litsea* sp.										+	+	+	3	7	0.2	Tree	Leaf
39		*Momordica balsamina*							+						1	20	0.7	Vine	Fruit
40		*Myrica esculenta* Buch.-Ham.ex D.Don.	+	+									+	+	4	66	2.2	Tree	Fruit, seed
41		*Myrsine capitellata* Wall		+						+	+	+		+	5	36	1.2	Tree	Fruit, seed, leaf
42		*Oplismenus* sp.		+		+		+	+	+		+		+	7	58	1.9	Grass	Flower, leaf, shoot
43		*Paspalum* sp.					+								1	4	0.1	Grass	Flower
44		*Peperomia tetraphylla*												+	1	1	0.0	Shrub	Stem
45		*Persea duthiei* (King ex Hook.f.) Kosterm				+									1	54	1.8	Tree	Fruit
46		*Persicara* sp.		+							+	+	+	+	5	104	3.4	Grass	Leaf
47		*Phragmites australis*							+	+					2	1	0.0	Herb	Leaf
48		*Pilea scripta* (Buch.-Ham. ex D. Don) Wedd.												+	1	4	0.1	Shrub	Leaf
49		*Piper mullesua*									+	+		+	3	104	3.4	Vine	Fruit
50		*Pleione humilis*												+	1	1	0.0	Orchid	Stem
51		*Polygonum* sp.							+						1	1	0.0	Grass	Flower
52		*Prunus cerasoides* D.Don	+	+			+							+	4	57	1.9	Tree	Fruit, seed, leaf, sap
53		*Prunus domestica*		+											1	27	0.9	Tree	Fruit
54		*Punica granatum* L.		+											1	1	0.0	Tree	Fruit
55		*Pyracantha* sp.					+								1	4	0.1	Tree	Fruit
56		*Pyrus pashia* Buch-Ham		+			+	+	+			+			5	61	2.0	Tree	Fruit
57		*Quercus semecarpifolia* Sm									+				1	8	0.3	Tree	Sap, bark
58		*Rhododendron* sp.												+	1	10	0.3	Tree	Flower
59		*Rubia manjith*								+	+			+	3	7	0.2	Herb	Leaf
60		*Rubus acuminatus*												+	1	16	0.5	Shrub	Leaf, stem
61		*Rubus ellipticus* sm.	+	+										+	3	34	1.1	Shrub	Fruit
62		*Saurauia napaulensis* DC.					+	+				+		+	4	10	0.3	Shrub	Fruit
63		*Schima wallichii* (D.C) Korth.	+	+								+		+	4	157	5.2	Tree	Fruit
64		*Smilax* sp.				+	+								2	5	0.2	Climber	Fruit
65		*Tagetse* sp.									+				1	1	0.0	Herb	Flower
66		*Tetrastigma serrulatum*												+	1	2	0.1	Climber	Fruit
67		*Thysanolaena* sp.										+			1	2	0.1	Grass	Stem
68		*Trifolium* sp.	+	+	+		+				+	+	+	+	8	579	19.0	Herb	Leaf
69		*Urtica dioica*	+	+						+			+	+	5	12	0.4	Shrub	Leaf
70		*Viburnum mullaha*									+				1	24	0.8	Shrub	Fruit
71		*Zea mays*					+	+							2	15	0.5	Grass	Fruit, stem
72		*Zizyphus incurva* Roxb.						+	+		+	+		+	5	68	2.2	Tree	Fruit, seed
Subtotal			16	20	12	3	17	16	11	10	22	25	17	38		2863	94.1		
	Others																		
		Moss									+				1	10	0.3		
		Mushroom				+	+	+							3	20	0.7		
		Insect and spider			+	+	+	+	+	+					6	39	1.3		
		liverworts												+	1	2	0.1		
		Soil					+		+		+	+	+		5	107	3.5		
Subtotal																178	5.8		
Total																3041	100.0		
Assamese macaque																			
1		*Acer oblongum*	+												1	4	0.1	Tree	Leaf, fruit
2		*Albizia procera* (Roxb.) Benth.								+	+				2	84	1.7	Tree	Leaf, fruit
3		*Albizia* sp.		+								+			2	7	0.1	Tree	Fruit, sap
4		*Ardisia macrocarpa*		+	+			+			+	+			6	16	0.3	Shrub	Leaf, fruit
5		*Bambuseae kunth*				+	+	+	+	+					5	5	0.1	Grass	Leaf, shoot
6		*Betula alnoides*								+		+	+	+	4	398	8.2	Tree	Leaf, fruit, flower
7		*Bombax ceiba*	+	+							+		+	+	5	99	2.0	Tree	Leaf, fruit, flower, shoot
8		*Castanopsis indica*							+						1	3	0.1	Tree	Leaf
9		*Castanopsis tribuloides*	+			+	+	+							4	21	0.4	Tree	Leaf, fruit
10		*Cautleya spicata*		+	+	+									3	23	0.5	Herb	Leaf, stem, flower, bark
11		*Celtis australis* L.	+	+				+	+						4	21	0.4	Tree	Leaf, fruit
12		*Choerospondias axillaris*						+	+		+				4	43	0.9	Tree	Leaf, fruit
13		*Entada phaseoloides*				+	+	+							3	11	0.2	Climber	Leaf, fruit, bark
14		*Eurya acuminata* DC.	+	+											2	2	0.0	Tree	Fruit
15		*Ficus lacor*	+	+	+	+									4	24	0.5	Tree	Flower, leaf, fruit
16		*Ficus religiosa*		+											1	5	0.1	Tree	Leaf, fruit
17		*Ficus semicordata*	+				+	+	+						4	21	0.4	Tree	Leaf, fruit, bark
18		*Ficus* sp.		+	+	+	+								4	8	0.2	Tree	Leaf, fruit
19		*Hedyotis scandens*	+	+		+	+	+	+						6	50	1.0	Herb	Leaf, stem, gum
20		*Helixanthera parasitica*	+				+		+	+	+	+	+	+	7	31	0.6	Parasite	Fruit, leaf
21		*Kingidium taenialis*							+						1	4	0.1	Herb	Fruit
22		*Lantana camara*	+	+	+	+	+	+	+	+	+	+	+	+	12	495	10.2	Shrub	Shoot, leaf, fruit
23		*Myrica esculenta* Buch.-Ham.ex D.Don.	+												1	3	0.1	Tree	Fruit
24		Orchid (unidentified)								+					1	3	0.1	Orchid	Leaf
25		*Prunus cerasoides* D.Don	+	+	+			+	+	+	+	+	+	+	9	255	5.3	Tree	Fruit, leaf, sap, flower
26		*Psidium* sp.							+						1	7	0.1	Tree	Leaf, fruit, bark
27		*Rhus* sp. (1)	+	+	+	+	+								5	67	1.4	Tree	Leaf, fruit
28		*Rhus* sp. (2)						+							1	1	0.0	Tree	Leaf
29		*Rhus succedanea*	+		+										2	2	0.0	Tree	Leaf
30		*Schima wallichii* (D.C) Korth.	+		+	+		+	+	+	+	+	+	+	11	586	12.1	Tree	Fruit, leaf, flower, bark,
31		*Smilax aspera*			+			+	+						3	5	0.1	Climber	Flower, fruit
32		*Smilax lanceifolia*							+						1	1	0.0	Climber	Leaf
33		*Toddalia asiatica*		+		+	+	+	+	+	+	+	+	+	10	85	1.8	Climber	Leaf, sap, fruit
34		*Trichosanthes tricuspidata*							+						1	3	0.1	Climber	Leaf
35		*Trichosanthes wallichiana*		+		+	+	+							4	32	0.7	Climber	Leaf, fruit, bark
36		*Viburnum mullaha*							+						1	2	0.0	Shrub	Fruit
37		*Zea mays*			+	+	+								3	61	1.3	Grass	Fruit
38		*Zizyphus incurva* Roxb.					+	+	+	+	+		+	+	7	492	10.1	Tree	Leaf, fruit, flower, bark
Subtotal			15	15	11	13	13	16	18	11	11	9	8	9		2980	61.4		
	Others																		
		Lichen	+			+	+								3	6	0.1		
		Mushroom	+	+	+	+	+	+	+	+	+	+	+	+	12	395	8.1		
		Insect and spider	+	+	+	+	+	+	+	+	+	+	+	+	12	1446	29.8		
		Soil		+			+			+		+		+	4	23	0.5		
Subtotal																1870	38.6		

“+”: macaques fed on the species during scan sampling

Provisioned foods and leftover fruits and vegetables were not included

**Table 2:** Additional food species of rhesus macaques during all occurrence sampling

No.	Species	Life	Parts eaten
1	*Ampelocissus sikkimensis* (M. A. Lawson)	Climber	Fruit
2	*Apois carnea*	Vine	Flower, leaf
3	*Bauhinia malabarica* Roxb	Tree	Leaf
4	*Crassocephalum crepidioides*	Herb	Stem, leaf
5	*Cyperus rotundus*	Grass	Flower, stem
6	*Daphne bholua*	Shrub	Stem
7	*Desmodium* sp.	Herb	Flower, leaf
8	*Flemingia macrophylla*	Shrub	Flower
9	*Hedychium* sp.	Shrub	Stem, flower
10	*Lindera pulcheriima* (Nees) Benth ex Hook. f.	Tree	Fruit, leaf
11	*Poa* sp.	Grass	Leaf
12	*Rabhidophora* sp.	Climber	Cone
13	*Rumex* sp.	Herb	Leaf
14	*Sedum* sp.	Herb	Leaf
15	*Shuteria involucrata* var. *glabrata* (Wall.)	Climber	Flower
16	*Solena heterophylla Lour*	Climber	Leaf

**Table 3:** Forms and eaten parts of food plants of rhesus and Assamese macaques

	Rhesus macaque	Assamese macaque
*Plant form*		
Tree	27	22
Herb	16	3
Shrub	20	3
Climber	9	6
Vine	3	0
Grass	12	2
Orchid	1	1
Total	88	37
*Parts eaten*		
Fruit and cone	31	20
Seed and pod	6	0
Flower	14	7
Leaf	35	25
Stem	8	1
Bark	1	3
Nector and sap	3	1
Whole plant	2	1
Total	100	58

Number of species on which macaques fed are shown

There were significant associations between the time of day and quality of food in both species (*χ*^2^ test, *df* = 3, *χ*^2^ = 111.58, *p* < 0.05 in Assamese macaques; *df* = 3, *χ*^2^ = 80.34, *p* < 0.05 in rhesus macaques). Both macaques more frequently fed on high-quality food than low-quality food before 11:00, although there were some inter-species differences after 11:00. Assamese macaques more frequently fed on high-quality food than low-quality food during 11:00–14:00, whereas dependency on high-quality after 14:00 was less. Comparably, rhesus macaques more frequently fed on high-quality food than low-quality food during 14:00–18:00, whereas dependency was less during 11:00–14:00.

There was no significant association between the age class (adult vs. sub-adult and others) and quality of food in Assamese macaques, but a relationship exist between quality of food and age class in rhesus macaques (*χ*^2^ test, *df* = 1, *χ*^2^ = 2.23, n.s. in Assamese macaque; *df* = 1, *χ*^2^ = 22.02, *p* < 0.05 in rhesus macaque). However, in both study groups, sub-adult females depended more on high-quality food (*χ*^2^ test, *df* = 1, *χ*^2^ = 5.7, *p* < 0.05 in Assamese macaques; *df* = 1, *χ*^2^ = 15.34 in rhesus macaques).

### Ranging

**Fig. 6:**
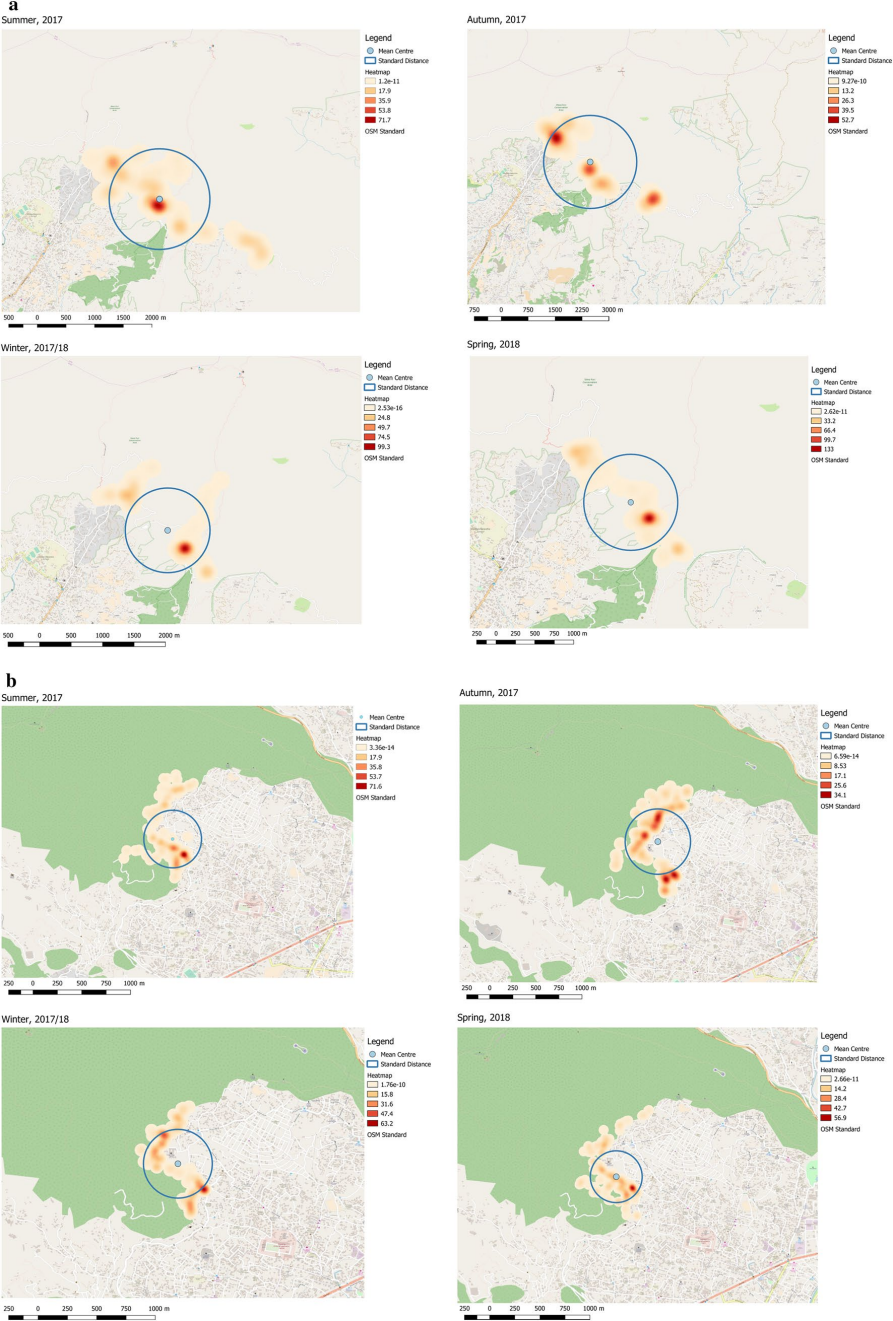
Heat maps and ranging patterns of rhesus macaques in the RN group and Assamese macaques in the AS group at Shivapuri-Nagarjun National Park during four seasons. **a** Rhesus macaque, **b** Assamese macaque

**Fig. 7:**
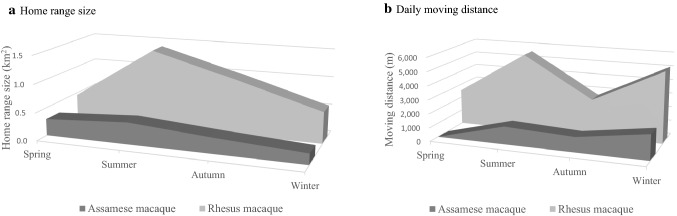
Home-range size and daily moving distance of rhesus macaques (larger area) in the RN group and Assamese macaques in the AS group. **a** Home-range size. **b** Moving distance. The distance of Assamese macaques in spring is shown as zero, because there was no data available

Assamese macaques were less mobile than rhesus macaques (Figs. 6 and 7). The yearly area covered by Assamese macaques in the AS group was 0.55 km^2^, whereas that covered by rhesus macaques in the RN group was 4.23 km^2^ (Fig. 6; Wilcoxon’s signed-rank test, *n* = 12, *z* = − 2.85, *p* < 0.05). Assamese macaques had a shorter daily moving distance (1555.5 m) than rhesus macaques (4036.0 m) (Mann–Whitney *U* test, *n*1 = 13, *n*2 = 13, *z* = − 3.16, *p* < 0.05). Assamese macaques performed 94.0% of their activities in trees, whereas rhesus macaques spent most of their time at ground level, with 58.5% of activities on the ground (*χ*^2^ test, *df* = 1, *χ*^2^ = 14,010.2, *p* < 0.05).

Assamese macaques covered greater area during summer (mean 0.39 km^2^), whereas least area during winter (mean 0.19 km^2^). Contrary, rhesus macaques covered greater area during summer (mean 1.41 km^2^) and least during spring season (mean 0.46 km^2^). Assamese macaques spent most of their time (94.4%) in trees in spring, and less time in trees in summer (89.5%), autumn (93.8%), and winter (94.0%). In contrast, rhesus macaques spent most of their time (63.1%) on the ground in spring and less time in summer (51.2%), autumn (54.5%), and winter (62.0%).

Assamese macaques spent most time in the trees throughout the day: before 08:00 (100%), during 08:00–11:00 (98.1%), 11:00–14:00 (95.1%), 14:00–17:00 (91.2%), and after 17:00 (87.6%), whereas rhesus macaques mostly engaged in activities on the ground (60.5% during 14:00–17:00; 58.8% during 11:00–14:00), and in the trees after 17:00 (58.5%) and* Pan troglodytes* 08:00–11:00 (51.6%) (*χ*^2^ test, *df* = 3, *χ*^2^ = 140.58, *p* < 0.05).

In Assamese macaques, adults spent less time in trees (93.1%), whereas other group members spent 94.7% of their time in trees. In rhesus macaques, adults and other group members spent 63.3 and 54.1% of their time on the ground, respectively (*χ*^2^ test, *df* = 1, *χ*^2^ = 24.1, *p* < 0.05 in Assamese macaques; *df* = 1, *χ*^2^ = 202.4, *p* < 0.05 in rhesus macaques). The proportions of time spent on the ground or in the trees did not differ significantly between males and females (*χ*^2^ test, *df* = 1, *χ*^2^ = 3.71, n.s. in Assamese macaques; *χ*^2^ = 1.33, n.s. in rhesus macaques).

## Discussion

The results of this study showed that Assamese and rhesus macaques utilize different habitats, perform different activities, and exhibit varying dietary dependence and ranging patterns. These findings corroborate our predictions and strongly indicate that large groups of different primate species establish non-overlapping territories, assuming that the primates have already undergone resource partitioning.

Among four daily activities (feeding, resting, moving, and social behavior), Assamese macaques spent most of their time feeding (30.7%), as reported by Justa et al. (2019), and less in social activities, whereas rhesus macaques spent most time in social activities and less in moving. A previous study found that Assamese macaques spent more time resting and less time feeding than rhesus macaques in limestone habitats, China (Zhou et al. 2014). However, a similar study on Assamese and rhesus macaques in the Western Himalaya, India, found that Assamese macaques spent more time in feeding (Justa et al. 2019), similar to our finding. Our observations indicated that Assamese macaques in the AS group showed ecological features similar to those of Assamese macaques in previous studies at SNNP and other sites. They spent 30.8% of their diurnal time in feeding, whereas Assamese macaques at Phu Khieo in Thailand spent 31–34% of their time in feeding (Schülke et al. 2011). Feeding time of Assamese macaques in a semi-provisioned group and a wild group at SNNP were 37 and 55%, respectively (Koirala et al. 2017). The differences between feeding activity found in Koirala et al. (2017) and present study may reflect differences in group size; however, feeding dependency may or may not be related to food abundance but to the desirability of the food (McConkey et al. 2002). Similarly, activity budgets within species are different between lactating and gestating females (Vasey 2005), between adults and juveniles (Janson and van Schaik 1993), and between males and females, caused by the variety of energy demand in each age–sex class. Our results showed significant differences in activity budget between males and females. Female rhesus macaques showed characteristic activities for energy accumulation through feeding, while males spent more time for social activities. Group dynamics must have resulted in such variation, as there were a large number of infants in the RN group, females are under pressure to accumulate and conserve energy, and, due to less number of resident male in the group, males are under pressure to release group tension and create stable group with less conflict through social activities. Contrary, Assamese macaque females as well as males showed pattern of energy accumulation through feeding and resting respectively, which might be due to their arboreal mode of living. Thus, variation in feeding activity across age classes can indicate group dynamic in their populations and species.

Diet of the two species varied markedly in this study; only 17 plant genera were shared between the two study groups. Rhesus macaques were dependent on more plant species than were Assamese macaques. Of the two major dietary components, plants and insects, Assamese macaques were predominantly folivores, as reported in previous studies (Justa et al. 2019; Zhou et al. 2014, 2018), followed by frugivory. Insects were another major dietary component for Assamese macaques, compared to rhesus macaques. However, it should be noted that diet preferences can differ between populations. Chalise (2003) and Huang et al. (2015) reported that Assamese macaques preferred flowers and fruits when their availability was dominant; nonetheless, these findings conflict with those of the present study and most previous studies. Feeding preferences of rhesus macaques in the present study included leaves, as reported by Goldstein and Richard (1989) and Tang et al. (2016), but unlike most findings on frugivory (Schülke et al. 2011; Zhou et al. 2014; Justa et al. 2019; Feeroz 2011). Nevertheless, without phenological information on food availability and distribution, our study cannot reliably address the factors of seasonality in their activity patterns, because fruit consumption is influenced by availability (Sengupta and Radhakrishna 2016) and activities are shaped by the environmental and physiological systems of the species (Ruslin et al. 2019).

Assamese macaques were dependent on fewer food items than rhesus macaques, which suggests that rhesus macaques were more prone to wandering due to opportunistic feeding and are more exploratory than Assamese macaques. The broad range of food items, along with seasonal changes in home range size and disbursement of dietary dependence may indicate that rhesus macaques employed a fallback food strategy in which they utilize alternative food items when the preferred food is unavailable (Marshall and Wrangham 2007). Such a generalist feeding behavior is believed to have played a major role in the ecology and evolution of primate groups. As our study groups share the same ancestor (Li et al. 2009), this type of food selection could have contributed to divergence in the primate lineage. This mechanism is thought to have had an influence on morphology, group size, and population density (Hanya et al. 2006; Matsumoto-Oda et al. 1998). Further, the Pianka index of niche overlap (0.5) was higher than that in a previous study of Assamese and rhesus macaques in China (Zhou et al. 2014; Singh et al. 2011; Sushma and Singh 2006). It has also been noted that, among sympatric primate resource sharing is high (Chapman and Pavelka 2005; Cords 1986). Since we performed analysis at genus level of vegetation, we expect niche overlap to be even lower at species level.

A closer observation in ranging patterns showed that total home range size of Assamese macaques in the AS group in SNNP was only 0.55 km^2^, whereas Assamese macaques at Phu Khieo, Thailand, used an area of 4.7 km^2^ (Schülke et al. 2011). Home ranges at Bhutan ranged between 3.2 and 5.4 km^2^ (Oi et al. 2016), and that in China was 0.53–0.65 km^2^ (Zhou et al. 2014). Differences in home range size can be attributed to variation in geography and vegetation, as folivores have smaller ranges than do frugivore primates (Clutton-Brock and Harvey 1977). Assamese macaques in Bhutan inhabited arid shrubby grasslands with low-productivity forests, whereas Assamese macaques in China inhabited a limestone seasonal rainforest, and those in Thailand inhabited a seasonal rainforest. We found that Assamese macaques daily travelled only 1.6 km, whereas rhesus macaques daily travelled 4.0 km. This behavioral difference may have been responsible for not only their feeding strategies but the variations in morphological characters and body size between the two species. Less moving conserves energy, which in turn reduces caloric expenditure, resulting in a stocky body build. Fleagle and Mittermeier (1980) reported that primate species with different body size shows variation in locomotor and postural activities. Even differences in body size within species correlated with differences in locomotor behavior (Doran 1993). Similarly, morphological differences, i.e., postcranial differences, have been reported to correlate with behavioral differences as illustrated by “suspensory hypothesis” (Susman 1979), stating that morphological differences between bonobos (*Pan paniscus*) and chimpanzees (*Pan troglodytes*) are a result of bonobos’ adaptation to forest dwelling and arboreal mode of living.

A notable difference between the two macaque species examined in this study is that the Assamese macaques were arboreal, whereas the rhesus macaques were terrestrial. Such vertical habitat partitioning has been reported in the grey langur (*Semnopithecus entellus*), lion-tailed macaque (*Macaca silenus*), and bonnet macaque (*Macaca radiata*) (Singh et al. 2011; Feeroz 2011; Hadi et al. 2012; Lahann 2008; Justa et al. 2019). Vertical stratification acts as a mechanism to reduce interspecific competition (Ganzhorn 1989; Hadi et al. 2012; Schwab and Ganzhorn 2004). Tendency of Assamese macaques to spend time higher in the tree canopy may be affected by several factors. Doran (1993) and Fleagle and Mittermeier (1980) suggested that larger primates climb trees more than smaller primates. Also, predation pressure may have pushed the primates to occupy different habitats, as leopards mostly prey on monkeys on the ground during the day (Zuberbühler and Jenny 2002; Shrestha and Thapa 2016). Additionally, predation pressure by leopards is positively associated with the abundance, body size, group size, and number of males per group of monkeys (Zuberbühler and Jenny 2002), along with differences in geographical conditions and food distributions between the two study sites. Further study is required to explore this preference.

To summarize, the two primate species within similar nearby forests exhibited dissimilarities in socioecological behavior. This may be a mechanism to reduce the degree of conflict and share resources between the two species, and this type of behavioral adaptation can ultimately form a basis for evolutionary change, as dietary needs of species have dissimilar implications for habitat use (Erinjery et al., 2019). The results of this study increased our understanding of behavioral flexibility and adaptability in non-human primates. The differences in diet and habitat use between the two macaque species represent behavioral patterns that enable their coexistence through resource partitioning and indicate that rhesus macaques are more abundant than Assamese macaques. Preferring a broader dietary niche can enhance the survival and sustenance of a species/group. Furthermore, this study provided valuable information on wild coexisting non-human primates; such information can be used to devise wildlife management plans.
